# Genotypic variability in patients with clinical diagnosis of Bartter syndrome type 3

**DOI:** 10.1038/s41598-023-38179-6

**Published:** 2023-08-03

**Authors:** Alejandro García-Castaño, Sara Gómez-Conde, Leire Gondra, María Herrero, Mireia Aguirre, Ana-Belén de la Hoz, Luis Castaño, Renaltube group, Fernando Santos, Helena Gil-Peña, Eliecer Coto, Vanessa Loredo, Flor Ángel Ordóñez, Julián Rodríguez, Eva Braga, Olaya Hernández, Rocío Fuente, Débora Claramunt, Víctor Manuel García-Nieto, Félix Claverie-Martín, Elena Ramos-Trujillo, Maria Isabel Luis-Yanes, Elizabeth Córdoba-Lanús, Ana Perdomo-Ramirez, Gloria Mura-Escorche, Luis Castaño, Leire Madariaga, Gustavo Pérez de Nanclares, Alejandro García-Castaño, Mireia Aguirre, Leire Gondra, María Herrero, Aníbal Aguayo, Nélida García-Pérez, Gema Ariceta, Anna Meseguer, Gerard Cantero, Virginia Cantos-Pastor, Elena Pérez-González, Pablo Bello-Gutiérrez, Leire Madariaga

**Affiliations:** 1 Biocruces Bizkaia Health Research Institute, Barakaldo, Spain; 2 CIBERDEM, CIBERER, Endo-ERN, Madrid, Spain; 3 Pediatric Department, University of the Basque Country UPV/EHU, Bizkaia, Spain; 4 Pediatric Nephrology Department, Cruces University Hospital, Plaza de Cruces, 48903 Barakaldo, Bizkaia Spain; 5 Hospital Universitario Central de Asturias, Oviedo, Spain; 6 Hospital Universitario Nuestra Señora de Candelaria, Santa Cruz de Tenerife, Spain; 7 Hospital Universitario Cruces, Barakaldo, Bizkaia Spain; 8 Hospital Universitario Vall d’Hebron, Barcelona, Spain; 9 Hospital Universitario Virgen Macarena, Sevilla, Spain; 10 Hospital Quirónsalud Valle del Henares, Torrejón de Ardoz, Spain

**Keywords:** Medical genetics, Acid, base, fluid, electrolyte disorders

## Abstract

Bartter syndrome (BS) is a salt-losing hereditary tubulopathy characterized by hypokalemic metabolic alkalosis with secondary hyperaldosteronism. Confirmatory molecular diagnosis may be difficult due to genetic heterogeneity and overlapping of clinical symptoms. The aim of our study was to describe the different molecular findings in patients with a clinical diagnosis of classic BS. We included 27 patients (26 families) with no identified pathogenic variants in *CLCNKB*. We used a customized Ion AmpliSeq Next-Generation Sequencing panel including 44 genes related to renal tubulopathies. We detected pathogenic or likely pathogenic variants in 12 patients (44%), reaching a conclusive genetic diagnosis. Variants in *SLC12A3* were found in 6 (Gitelman syndrome). Median age at diagnosis was 14.6 years (range 0.1–31), with no history of prematurity or polyhydramnios. Serum magnesium level was low in 2 patients (33%) but urinary calcium excretion was normal or low in all, with no nephrocalcinosis. Variants in *SLC12A1* were found in 3 (BS type 1); and in *KCNJ1* in 1 (BS type 2). These patients had a history of polyhydramnios in 3 (75%), and the mean gestational age was 34.2 weeks (SD 1.7). The median age at diagnosis was 1.8 years (range 0.1–6). Chronic kidney disease and nephrocalcinosis were present in 1 (25%) and 3 (75%) patients, respectively. A variant in *CLCN5* was found in one patient (Dent disease), and in *NR3C2* in another patient (Geller syndrome). Genetic diagnosis of BS is heterogeneous as different tubulopathies can present with a similar clinical picture. The use of gene panels in these diseases becomes more efficient than the study gene by gene with Sanger sequencing.

## Introduction

Bartter syndrome (BS) type 3 or classic BS is a salt-losing hereditary tubulopathy caused by molecular defects in the *CLCNKB* gene (MIM * 602023), coding for the basolateral chloride channel ClC-Kb. It is characterized by renal salt wasting, hypokalemic metabolic alkalosis and secondary hyperaldosteronism with normal blood pressure^1^.

Large phenotypic variability has been described in patients with BS type 3 due in part to the expression of the chloride channel ClC-Kb in several segments of the nephron and to the possible compensatory function of the ClC-Ka channel, which shares a high homology with the ClC-Kb channel^2^. Although the first reports of classic BS syndrome described patients with infant or childhood onset, severe hypokalemia and hypercalciuria as clinical hallmarks, a significant number of them can present with an antenatal/neonatal onset (typical of BS type 1,2 & 4) or with a Gitelman-like onset^3^.

Other salt-losing tubulopathies can also mimic classic BS. These usually share hypokalemia as a common biochemical sign, which is secondary to the activation of the renin-angiotensin aldosterone system due to excessive sodium and water loss. However, the clinical presentation (age and severity) and other biochemical signs have classically been thought to be specific of each subtype of salt-losing tubulopathy. In accordance with this, Gitelman syndrome due to mutations in the *SLC12A3* gene (MIM * 600968) encoding for the NaCl cotrasporter in the distal convoluted tubule usually has an adulthood onset with an incidental discovery of mild hypokalemia and hypomagnesemia with hypocalciuria. However, it occasionally has a childhood onset with a more severe phenotype^4,5^. Antenatal/neonatal BS is the leading phenotype in BS type 1, 2 & 4, secondary to mutations in the *SLC12A1* (Na–K-2Cl Cotransporter, MIM * 600839), *KCNJ1* (Inwardly Rectifying K + Channel, MIM * 600359) and *BSND* (Barttin CLCNK Type Accessory Beta Subunit, MIM * 606412) genes^6^, respectively. But again, these can also present with normal neonatal phenotype and a later onset, mimicking classic BS^7,8^.

Due to the genotypic heterogeneity of these salt-losing tubulopathies, the approach to the molecular diagnosis with a targeted next generation sequencing panel is particularly useful. This enables the study of all the implicated genes at the same time.

The aim of our study was to describe the molecular findings in a cohort of patients with a clinical diagnosis of classic BS but no identified pathogenic variants in the *CLCNKB* gene, using a targeted sequencing panel including all the genes related to phenotypically similar salt-losing tubulopathies described to date.

## Methods

### Ethics statement

The study was approved by the Ethics Committee for Clinical Research of Euskadi (CEIC-E. Internal code PI2017118). Written informed consent was obtained from each patient and/or minors’ legal tutors before inclusion in the study. The research was carried out in accordance with the Declaration of Helsinki on human experimentation of the World Medical Association.

### Patients

The study included 27 patients from 26 unrelated families, with a clinical diagnosis of classic BS. An adult or a pediatric nephrologist in the referral hospital made the initial diagnosis. The cohort was mostly pediatric (18 patients [67%] were younger than 16 years at the time of initial clinical diagnosis) and the clinical criteria for the diagnosis necessarily included: (i) Renal salt wasting; (ii) Hypokalemic metabolic alkalosis; (iii) Absence of other salt wasting non-renal pathology.

These 27 patients came from an original cohort of 74 patients referred to our laboratory for the study of the *CLCNKB* gene between 2003 and 2016. The analysis of this gene was initially done with traditional Sanger sequencing for the detection of point mutations, and MLPA (Multiplex Ligation Dependent Probe Amplification) for the detection of large rearrangements, as described previously^9^. Pathogenic variants in this gene were found in 47 patients, thus, a diagnosis of BS type 3 was retained. These patients were excluded from the present study.

### DNA analysis

Extraction and purification of genomic DNA from peripheral blood leukocytes was performed according to the manufacturer’s instructions (MagPurix Blood DNA Extraction Kit 200. Zinexts Life Science Corp., New Taipei City, Taiwan, R.O.C.).

One customized Ion AmpliSeq Next-Generation Sequencing gene panel including 44 genes of interest was designed using the computer tool Ion AmpliSeq Designer (Thermo Fisher Scientific). These included the exonic regions, the 5' and 3' untranslated regions (UTRs) and the flanking intronic sequences of all the renal salt-wasting tubulopathy-related genes (*SLC12A1, KCNJ1, BSND, CLCNKB, CLCNKA, CASR, MAGED2*, *KCNJ10*, *SLC12A3, SCNN1B, SCNN1G, SCN4A, SCNN1A, NR3C2, WNK4, WNK1, CUL3, KLHL3*) and other genes whose mutations are associated with different tubulopathies (*ATP6V1B1, CA2, SLC4A4, SLC4A1, ATP6V0A4, AVPR2, AQP2, CLCN5, OCRL1, HNF1B, KCNA1, CLDN16, CLDN19, TRPM6, FXYD2, EGF, CNNM2, DMP1, FGF23, SLC34A3, PHEX, SLC34A1, SLC9A3R1, ENPP1, GNA11, AP2S1*). Library preparation was done according to the manufacturer’s instructions using Ion AmpliSeq Library Kit 2.0 (Thermo Fisher Scientific) followed by sequencing by the Ion GeneStudio S5 Plus (Thermo Fisher Scientific). Variants were filtered to include only those with a p-value < 0.001, and a Minor Allele Frequency (MAF) < 1% in 1000 Genomes Browser, Exome Aggregation Consortium (ExAC) and ESP Exome Variant Server (ESP). Not appropriately covered amplicons (< 20 × fold) and candidate variants were assessed by polymerase chain reaction (PCR) followed by Sanger sequencing.

### Copy number variation (CNV) analysis

In order to detect potential gross allelic deletions or duplications in the *SLC12A3* gene, a commercially available MLPA kit, SALSA MLPA probemix P136-B4 (MRC Holland, Amsterdam, The Netherlands) was used.

Variants were named according to the Human Genome Variation Society guidelines, and then classified following the American College of Medical Genetics (ACMG) guidelines^10^.

## Results

In our cohort of 27 patients, we detected pathogenic or likely pathogenic variants that led to a confirmatory genetic diagnosis in 12 patients, thus 44% of the studied cohort, encompassing five different tubulopathies. Of the five distinct disorders detected, three genetic diagnoses corresponded to 37% of the cohort: Gitelman syndrome (6 cases), BS type 1 (3 cases), and BS type 2 (1 case). Additional diagnoses were made in a smaller proportion: one patient (SOR0118) had a variant in the *CLCN5* gene (MIM * 300,008). Pathogenic variants in this gene are associated with Dent disease type 1 (MIM # 300009), which is characterized by a proximal renal tubular defect, hypercalciuria, nephrocalcinosis and progressive kidney disease^11^. Finally, one patient (SOR0085) had a variant in the *NR3C2* gene (MIM * 600983). Pathogenic activating variants in this gene are associated with Geller syndrome or Early-Onset Hypertension with Exacerbation in Pregnancy (MIM # 605115)^12^. Details of the molecular and clinical characteristics of these patients are summarized in Tables 1 and 2, respectively.

**Table 1:** Molecular characteristics of patients with candidate variants in the described genes.

Patient	Gene	Mutation(s)*	Status	Inheritance pattern	Final diagnosis	References
SOR0021	*SLC12A3*	c.[1924C > T];[2576 T > C] p.[(Arg642Cys)];[Leu859Pro]	Compound heterozygous	Parents not studied	Gitelman syndrome	^13^/^14^
SOR0077	*SLC12A3*	c.[2542G > A];[2981G > A] p.[(Asp848Asn)];[Cys994Tyr]	Compound heterozygous	Parents: heterozygous carriers	Gitelman syndrome	^15^/^16^
SOR0087	*SLC12A3*	c.[2576 T > C]; p.[Leu859Pro]	Homozygous	Parents: heterozygous carriers	Gitelman syndrome	^14^
SOR0095	*SLC12A3*	c.[1928C > T];[2576 T > C] p.[(Pro643Leu)];[Leu859Pro]	Compound heterozygous	Parents: heterozygous carriers	Gitelman syndrome	^14^/^17^
SOR0119	*SLC12A3*	c.[1180 + 1G > T];p.[?]	Homozygous	Parents not studied	Gitelman syndrome	^18^
SOR0133	*SLC12A3*	c.[2576 T > C];[2981G > A] p.[Leu859Pro];[Cys994Tyr]	Compound heterozygous	Father: heterozygoyus carrier, mother unknown	Gitelman syndrome	^14^/^16^
SOR0079	*SLC12A1*	c.[223C > T];[2281C > T] p.[(Gln75*)];[(Arg761*)]	Compound heterozygous	Parents: heterozygous carriers	BS type 1	rs1048935147†/^6^
SOR0082	*SLC12A1*	**c.[596G > A]**;[1547C > A] **p.[(Arg199His)]**;[(Pro516His)]	Compound heterozygous	Parents not studied	BS type 1	**This study**/rs556137575†
SOR0098	*SLC12A1*	c.[511C > T];[1523C > A] p.[(Gln171*)];[(Ala508Asp)]	Compound heterozygous	Parents not studied	BS type 1	**This study**/**This study**
SOR0140	*KCNJ1*	c.[917C > T]; p.[(Ala306Val)]	Homozygous	Parents: heterozygous carriers	BS type 2	rs747718912†
SOR0118	*CLCN5*	**c.842A > G p.(Glu281Gly)**	Hemizygous	Mother: heterozygous carrier	Dent disease	**This study**
SOR0085	*NR3C2*	c.2852C > T p.(Ala951Val)	Heterozygous	Mother: heterozygous carrier	MR alteration	rs547308323†

Mutations marked in bold have not been reported to date.

*MR* mineralocorticoid receptor.

*Numbering is according to DNA sequence (Ensembl transcript *SLC12A3* gene: ENST00000438926.6; transcript *SLC12A1* gene: ENST00000380993.8; transcript *KCNJ1* gene: ENST00000392664.2; transcript *CLCN5* gene: ENST00000376091.8; transcript *NR3C2* gene: ENST00000358102.8).

^†^https://www.ncbi.nlm.nih.gov/.

**Table 2:** Clinical characteristics of patients with candidate variants in the described genes.

Patient	Molecular diagnosis	Amniotyc fluid	Gestational age (weeks)	Age at diagnosis (years)	Blood Pressure (mmHg)	Plasma Na + (mEq/L)^1^	Plasma K + (mEq/L)^2^	Plasma Cl − (mEq/L)^3^	Plasma Mg + (mg/dL)^4^	UCa2 + /Cr (mg/mg)^5^	Renal US	CKD stage
SOR0021	GS	–	–	3	–	–	< 3.5	–	1.71–2.5	0.05–0.2	Normal	0
SOR0077	GS	Normal	40	4	90/60	135	2.4	91	1.5	0.06	Normal	0
SOR0087	GS	–	–	24	116/76	139	3.2	–	1.7	< 0.05	Normal	0
SOR0095	GS	Normal	–	26	110/70	138	2.3	94	1.8	0.03	Normal	2
SOR0119	GS	–	–	0.1	–	< 135	–	–	1.71–2.5	0.05–0.2	Normal	0
SOR0133	GS	–	–	31	Normal	134	3.5	92	1.71–2.5	0.05	Normal	0
SOR0079	BS 1	Polyhydramnios	34	0.1	68/41	132	3	86	2.7	0.45	Nephrocalcinosis	0
SOR0082	BS 1	Polyhydramnios	35	0.16	100/50	134	2.9	–	–	–	Normal	0
SOR0098	BS 1	Normal	36	6	Normal	140	2.8	86	2.4	0.4	Nephrocalcinosis	3
SOR0140	BS 2	Polyhydramnios	32	1	Normal	139	3.7	96	2.7	0.34	Nephrocalcinosis	0
SOR0118	Dent disease	Normal	40	2	Normal	140	3.2	99	1.2	0.33	Normal	2
SOR0085	MR alteration	Normal	26	0.1	Normal	133	3	89	–	–	Nephrocalcinosis	0

*GS* Gitelman síndrome, *BS 1* Bartter syndrome type 1, *BS 2* Bartter syndrome type 2, *MR alteration* Mineralocorticoid receptor alteration, *CKD* Chronic kidney disease, *Renal US* renal ultrasound.

–Unavailable data.

^1^Plasma Na + level, normal range: 135–145 mEq/L.

^2^Plasma K + level, normal range: 3.5–5.2 mEq/L.

^3^Plasma Cl − level, normal range: 96–106 mEq/L.

^4^Plasma Mg + level, normal range: 1.71–2.5 mg/dL.

^5^UCa2 + /Cr: urinary calcium to creatinine ratio, normal range: 0.05–0.2.

Gitelman syndrome represents the most frequent tubulopathy diagnosed in this cohort: 6 patients (22%) carried pathogenic variants in *SLC12A3* in the homozygous or compound heterozygous state, and satisfied criteria for a conclusive diagnosis of Gitelman syndrome (Table 1). Among these patients, we found five missense and one splicing variant in intron 9 (Table 1). These variants have all been reported previously in the literature as pathogenic. In four cases, it was possible to assess the pattern of inheritance through the analysis of one or both parents, who were all heterozygous carriers of one of the pathogenic variants. Regarding the clinical findings in these patients with Gitelman syndrome, the median age at clinical diagnosis was 14.6 years (range 0.1–31). No history of polyhydramnios or prematurity was recalled. Serum magnesium levels were low (≤ 1.7 mg/dL) in 2 of the 6 patients (33%) and renal excretion of calcium was normal or low in all cases. One of the patients had a chronic kidney disease (CKD) stage 2 at the time of diagnosis (estimated glomerular filtration rate (eGFR) 80 ml/min/1.73m^2^). Renal ultrasound did not show nephrocalcinosis in any case.

BS type 1 represents 11% of the cohort (3 of 27): patients SOR0079, SOR0082 and SOR0098 carried two pathogenic or likely pathogenic variants in compound heterozygosity in the *SLC12A1* gene, thus satisfying criteria for a conclusive diagnosis of Bartter syndrome type 1. We detected 6 variants in the *SLC12A1* gene, three of which have not been reported previously in the literature (Table 1). According to ACMG-AMP guidelines, these three novel variants are classified as likely pathogenic. Regarding the remaining 3 variants, two of them have been previously reported as pathogenic (p.(Arg761*) and p.(Gln75*)) and the last one (p.(Pro516His)) is classified as likely pathogenic according to ACMG-AMP guidelines (Table 3). It was possible to assess the pattern of inheritance only in case SOR0079: parents’ genetic study revealed that they were heterozygous carriers of one pathogenic variant each.

**Table 3:** Classification of candidate variants according to ACMG-AMP guidelines (using the Sequence Variant Interpretation recommendations).

Gene	Nomenclature cDNA*	Nomenclature protein*	Selected criteria	Variant class
*SLC12A1*	c.596G > A	p.(Arg199His)	PM2_Moderate: Absent from controls in Exome Sequencing Project, 1000 Genomes Project, or Exome Aggregation Consortium (ExAC). PP3_Strong: Multiple lines of computational evidence support a deleterious effect on the gene or gene product (conservation, evolutionary, splicing impact, etc.). PM5_Supporting: Different amino acid change as a known pathogenic variant	Likely pathogenic
	c.1547C > A	p.(Pro516His)	PM2_Moderate: Low frequency in ExAC (1/121046) and GnomAD-Exomes (1/250314). PP3_Strong: Multiple lines of computational evidence support a deleterious effect on the gene or gene product (conservation, evolutionary, splicing impact, etc.)	Likely pathogenic
	c.511C > T	p.(Gln171*)	PVS1_Very Strong: Null variant (nonsense) in a gene where loss of function (LOF) is a known mechanism of disease. PM2_Moderate: Absent from controls in Exome Sequencing Project, 1000 Genomes Project, or Exome Aggregation Consortium (ExAC)	Likely pathogenic
	c.1523C > A	p.(Ala508Asp)	PM2_Moderate: Absent from controls in Exome Sequencing Project, 1000 Genomes Project, or Exome Aggregation Consortium (ExAC). PM3: For recessive disorders, detected in trans with a pathogenic variant. PM5_Moderate: Novel missense change at an amino acid residue where a different missense change determined to be pathogenic has been seen before (Ala508Thr). PP3_Strong: Multiple lines of computational evidence support a deleterious effect on the gene or gene product (conservation, evolutionary, splicing impact, etc.)	Likely pathogenic
*KCNJ1*	c.917C > T	p.(Ala306Val)	PM2_Moderate: Low frequency in ExAC (1/121382) and GnomAD-Exomes (1/251192). PM3_Supporting: For recessive disorders, detected in trans with a likely pathogenic variant. PM5_Supporting: Novel missense change at an amino acid residue where a different missense change determined to be pathogenic has been seen before (p.Ala306Thr). PP2_Supporting: Missense variant in a gene that has a low rate of benign missense variation and in which missense variants are a common mechanism of disease. PP3_Supporting: Multiple lines of computational evidence support a deleterious effect on the gene or gene product (conservation, evolutionary, splicing impact, etc.)	Likely pathogenic
*CLCN5*	c.842A > G	p.(Glu281Gly)	PM1_Moderate: Located in a well-established functional domain (The change affects an amino acid (281) that is important to channel functionality since is involved in linking H + and Cl − transport (UniProt: P51795)). PM2_Moderate: Absent from controls in Exome Sequencing Project, 1000 Genomes Project, or Exome Aggregation Consortium (ExAC). PP2_Supporting: Missense variant in a gene that has a low rate of benign missense variation and in which missense variants are a common mechanism of disease. PP3_Strong: Multiple lines of computational evidence support a deleterious effect on the gene or gene product (conservation, evolutionary, splicing impact, etc.)	Likely pathogenic
*NR3C2*	c.2852C > T	p.(Ala951Val)	PM1_Moderate: Located in a well-established functional domain (NR LBD). PP1_ Moderate: Co-segregation with disease in multiple affected family members in a gene definitively known to cause the disease. PP2_Supporting: Missense variant in a gene that has a low rate of benign missense variation and in which missense variants are a common mechanism of disease. PP3_Supporting: Multiple lines of computational evidence support a deleterious effect on the gene or gene product (conservation, evolutionary, splicing impact, etc.)	Likely pathogenic

*NR LBD* Nuclear receptor ligand-binding domain, *PVS1* Very strong evidence of pathogenicity, *PM* Moderate evidence of pathogenicity, *PP* Supporting evidence of pathogenicity, *BS* Strong evidence of benign impact.

*Numbering is according to DNA sequence (Ensembl transcript *SLC12A3* gene: ENST00000438926.6; transcript *SLC12A1* gene: ENST00000380993.8; transcript *KCNJ1* gene: ENST00000392664.2; transcript *CLCN5* gene: ENST00000376091.8; transcript *NR3C2* gene: ENST00000358102.8).

BS type 2 represents 4% of the cohort (1 of 27 patients): patient SOR0140 satisfied criteria for a conclusive diagnosis of BS type 2, as he was a homozygous carrier of the variant p.Ala306Val in the *KCNJ1* gene. It was possible to assess the pattern of inheritance in this case and both parents were heterozygous carriers of the same variant.

Regarding the clinical characteristics of the 4 patients with BS type 1 and 2, a history of polyhydramnios was recalled in 3 patients (75%), with a mean gestational age at birth of 34.2 weeks (SD 1.7). The median age at clinical diagnosis was 1.8 years (range 0.1–6). One of the 4 patients (25%) had a CKD stage 3 at the time of diagnosis (eGFR 55 ml/min/1.73m^2^). The mean level of potassium in plasma was 3.1 mEq/L (SD 0.4) at diagnosis. Nephrocalcinosis was present in 3 of the 4 patients (75%).

As for the diagnosis of Dent disease, patient SOR0118 had a likely pathogenic variant in the *CLCN5* gene in the hemizygous state, according to the expected transmission pattern. To our knowledge, this variant has not been previously reported in the literature and it is classified as likely pathogenic according to ACMG-AMP guidelines (Table 3). The mother of this patient was a heterozygous carrier of the same variant. Regarding the clinical characteristics, he had no history of polyhydramnios and was born at term. He had a mild CKD (eGFR 70–80 ml/min/1.73m^2^) from the first years of life (no history of acute dehydration was recalled) and low serum potassium and magnesium levels at diagnosis (3.2 mEq/L and 1.2 mg/dL, respectively). A mild hypercalciuria (urinary calcium to creatinine ratio: 0.3 mg/mg of creatinine, normal value < 0.2 mg/mg of creatinine) was present but renal ultrasound did not show nephrocalcinosis. He also presented with polyuria (2.3 L/m^2^, polyuria defined as urine output > 2L/m^2^) at diagnosis. Further clinical investigation in this patient after molecular confirmatory diagnosis, demonstrated severe low molecular weight proteinuria. At last follow-up, his CKD had progressed and he had developed hypophosphatemia too.

Finally, in patient SOR0085 a probably activating variant was found in the *NR3C2* gene, coding for the mineralocorticoid receptor. This variant is classified as likely pathogenic (Table 3). This variant was also present in the mother in the heterozygous state. Regarding clinical history, this patient was born prematurely at 26 weeks of gestation due to preeclampsia with severe and uncontrolled hypertension in the mother. He showed hyponatremia and hypokalemia, with metabolic alkalosis and normal blood pressure during the first years of life, and received potassium supplements. He progressively recovered a normal tubular function and had no need of supplements. No data was available about the levels of aldosterone at that time.

## Discussion

In this study, we analyzed the prevalence of mutations in genes related to salt-losing and other different tubulopathies in a cohort of patients with a clinical diagnosis of classic BS and no identified pathogenic variants in the *CLCNKB* gene. We described the molecular findings in several genes in these patients and the clinical characteristics associated with each different final genetic diagnosis. Therefore, in this study we report on the advantage of the use of a targeted –sequencing panel of genes to optimize the molecular diagnosis of patients with salt-losing tubulopathies.

In our initial cohort of 74 patients with a clinical diagnosis of classic BS, molecular confirmatory diagnosis of BS type 3 was initially reached in 47 patients (63%). Using a targeted-gene panel approach, pathogenic variants in different genes were discovered in 12 patients, leading to a conclusive genetic confirmation. In all cases, the genetic findings corresponded to a different tubulopathy that may phenocopy the initial clinically suspected BS type 3. In summary, using a targeted sequencing panel including all the genes related to phenotypically similar salt-losing tubulopathies, a molecular confirmatory diagnosis was reached in 80% of the initial cohort. A recent study using a targeted-gene panel approach in patients with a clinical diagnosis of primary renal tubulopathy (childhood-onset) showed a high diagnostic yield of 64%^19^. However, a similar study in adults showed a much lower diagnostic yield of 26%. This is probably due to the more complex phenotypes in adults with renal tubulopathies, especially in those who develop CKD, and the higher rate of acquired tubular dysfunction compared to children^20^. In our cohort, a selection bias of excluding patients with *CLCNKB* variants and the lower number of patients included, probably explain the higher diagnostic yield reached.

Half of the patients (6/12) where a conclusive genetic confirmation was reached had Gitelman syndrome. This is not surprising as previously reported large series of patients with a molecular diagnosis of BS type 3 or Gitelman syndrome have shown how both phenotypes can overlap^3,4^. Although usually less severe, hypokalemia in Gitelman syndrome, as in BS, is secondary to the activation of the renin-angiotensine aldosterone system, which increases the secretion of potassium in the collecting duct. Hypomagnesemia is thought to be linked with the sodium waste in the distal convoluted tubule, where Mg2 + reabsorption is tightly regulated^21^. Patients with Gitelman syndrome in our series were clinically diagnosed in the first years of life, and hypomagnesemia was found in only one third. Previous case reports have also shown how patients harboring mutations in *SLC12A3* can present with severe salt-losing related symptoms and be diagnosed early in childhood^5,22^. In addition, although hypomagnesemia was initially thought to be a cardinal sign in Gitelman syndrome, normal magnesemia has been reported in some patients^23^. As long-term prognosis of renal function is probably worse in patients with classic BS rather than Gitelman syndrome^3^, and complications may be different in both entities, the molecular confirmation in these patients is important for the long-term follow-up.

Patients with a molecular diagnosis of BS type 1 & 2 were characteristically younger at clinical diagnosis (< 2 years), and renal function was more frequently affected at diagnosis than what is usually described in patients with classic BS^3,24^. Nephrocalcinosis was also present at diagnosis in most of these patients, as described in other series^6^. Both the prematurity and the frequent nephrocalcinosis could have contributed to a bigger rate of CKD in these patients.

Regarding patient SOR0118 with a diagnosis of Dent disease, this diagnosis was clinically important as it leads to a variation in the information given to the patient about the inheritance pattern and the natural evolution of the disease: Dent disease is inherited as an X-linked recessive trait, and up to 50% of the patients develop CKD in the 4th to 5th decade of life^3,25^. This patient presented with a hypokalemic metabolic alkalosis, a typical feature of Bartter syndrome which has occasionally been observed in patients with Dent disease^26^. It is probably secondary to the activation of the renin-angiotensine aldosterone system that occurred in response to water and salt loss, and could predict a worse long-term outcome of renal function^26^. Further clinical investigation in this patient after molecular confirmatory diagnosis, demonstrated severe low molecular weight proteinuria and other features of Fanconi syndrome such as renal phosphate loss, which are classic findings in Dent disease^27^.

The variant in *NR3C2* found in patient SOR0085 is thought to be an activating mutation that alters the interaction between the ligand and mineralocorticoid receptor^28^. Similar variants in the same nuclear receptor ligand-binding domain have been described in women with severe hypertension during pregnancy, due to the abnormal activation of the MR by progesterone, whose levels are typically elevated during pregnancy^12^. These activating variants have also been described in male patients with familial dominant forms of hypertension developing from the first decades of life, with suppressed renin and aldosterone^12,29^. This may be associated with the activation of the MR by cortisol and corticosterone: in contrast with their low affinity for the wildtype MR, these steroids bind the mutant receptor with high affinity^30^. In our case, it is possible that both the activation of the MR and the renal tubulopathy associated with the extreme prematurity could have contributed to the clinical findings (metabolic alkalosis with hypokalemia, medullary nephrocalcinosis) and to the spontaneous resolution of the dyselectrolitemia over time. At last follow-up, the patient was 11 years old and had normal blood pressure, no dyselectrolitemia and normal renin and aldosterone levels. This finding was very important for the family as the mother could develop again severe hypertension in future pregnancies with premature delivery and other associated problems, and the child could develop hypertension within time due to this activating mutation and should be followed up for this reason^29,31^.

Finally, in 15 patients we did not reach a confirmatory molecular diagnosis. Some genes that have recently been described in patients with salt-losing phenotypes were not included in our gene panel and could explain this to some degree^32^. First, pathogenic variants in the *KCNJ16* gene, that encodes the K^+^-channel Kir5.1, have been recently described in patients with Gitelman-like syndrome^33^. Second, mitochondrial DNA variants in *MT-TI* and *MT-TF* were demonstrated to be a cause of Gitelman-like syndrome in a large European collaboration study^34^. Third, activating variants in the *RRAGD* gene, encoding a small Rag GTPase, have been shown in patients presenting with hypomagnesemia, hypokalemia, metabolic alkalosis and a severe cardiomyopathy in some cases^35^. Finally, the referral nephrologist could have incorrectly diagnosed some patients, especially those clinically diagnosed in the adult age, as a primary tubulopathy (Bartter syndrome type 3).

With the development of the technology of next generation sequencing, the use of gene panels for the study of diseases with genotypic variability such as salt wasting-tubulopathies becomes more cost-effective that the study of gene by gene with traditional Sanger sequencing, or whole exome and whole genome sequencing, which require more challenging bioinformatic support. In addition, the use of panels with selected genes for specific diseases provides the advantage of achieving high coverage of genes of interest at lower costs compared to whole exome or genome sequencing approaches^19^. The molecular confirmation of these tubulopathies enables genetic counselling for future pregnancies, enhances adequate treatment and facilitates provision of a more accurate information to the patient and family about the possible complications and the natural course of the disease.

## Data Availability

The datasets used and/or analysed during the current study available from the corresponding author on reasonable request.
